# P-1337. Trends in the co-carriage of multiple carbapenemases among clinical Enterobacterales isolates and the in vitro activity of aztreonam-avibactam, ATLAS 2019-2023

**DOI:** 10.1093/ofid/ofaf695.1525

**Published:** 2026-01-11

**Authors:** Mark Estabrook, Julie Dickson, Gregory Stone, Katherine Perez, Daniel F Sahm

**Affiliations:** IHMA, Schaumburg, IL; IHMA, Schaumburg, IL; Pfizer, Inc., Groton, Connecticut; Pfizer, Inc., Groton, Connecticut; IHMA, Schaumburg, IL

## Abstract

**Background:**

Aztreonam-avibactam (ATM-AVI) is a β-lactam/β-lactamase inhibitor combination to treat infections caused by Gram-negative organisms, particularly those carrying metallo-β-lactamases (MBLs) and other β-lactamases. Aztreonam is stable to hydrolysis by MBLs and avibactam inhibits Class A, C, and some Class D enzymes. We examined ATM-AVI activity against Enterobacterales isolates producing one or more carbapenemase and the frequency of co-production of carbapenemases among isolates collected as a part of the ATLAS global surveillance program (2019-2023).

**Figure:**
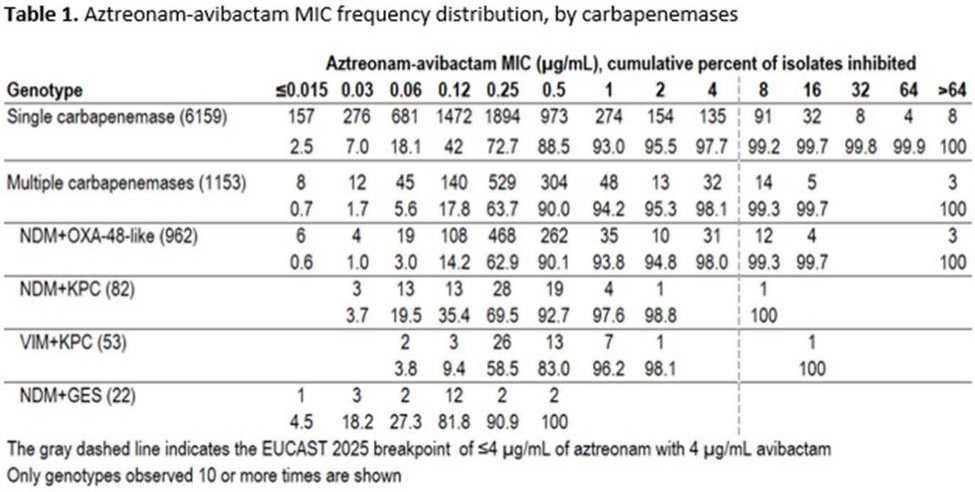

**Figure:**
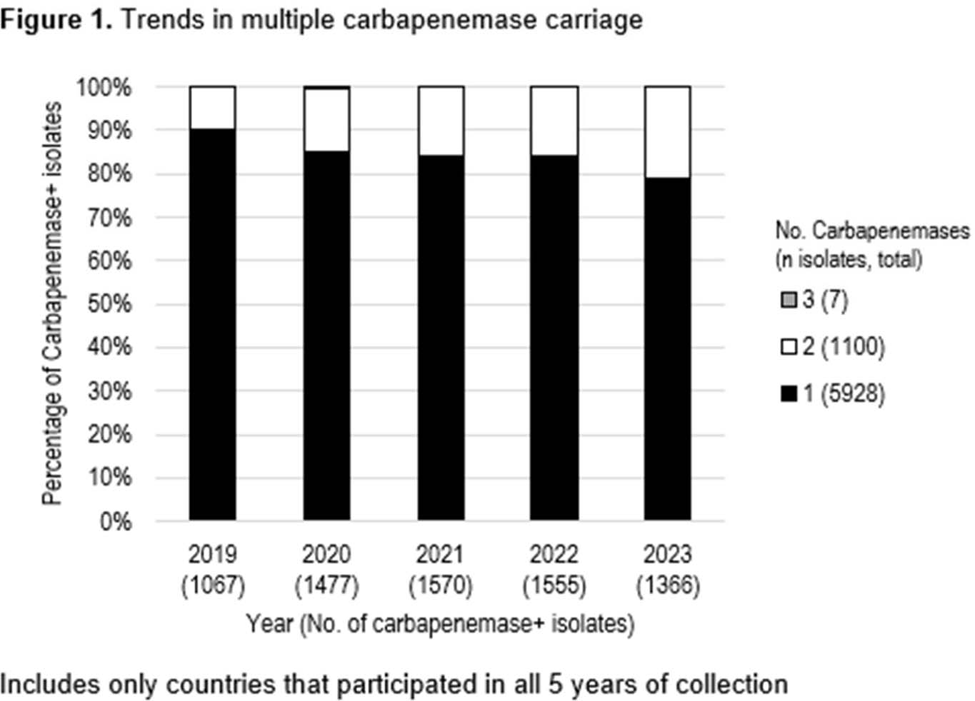

**Methods:**

88,196 isolates from 226 medical centers in 56 countries (excluding mainland China, Canada, and the USA) were collected and tested for susceptibility using the broth microdilution method according to CLSI guidelines. Analysis was performed with CLSI 2024 breakpoints. Isolates testing with meropenem MIC values >1 µg/mL or *Escherichia coli, Klebsiella pneumoniae, K. oxytoca,* or *Proteus mirabilis* isolates testing with ceftazidime and/or aztreonam MIC values >2 µg/mL were screened for β-lactamase genes by PCR, which were sequenced when identified.

**Figure:**
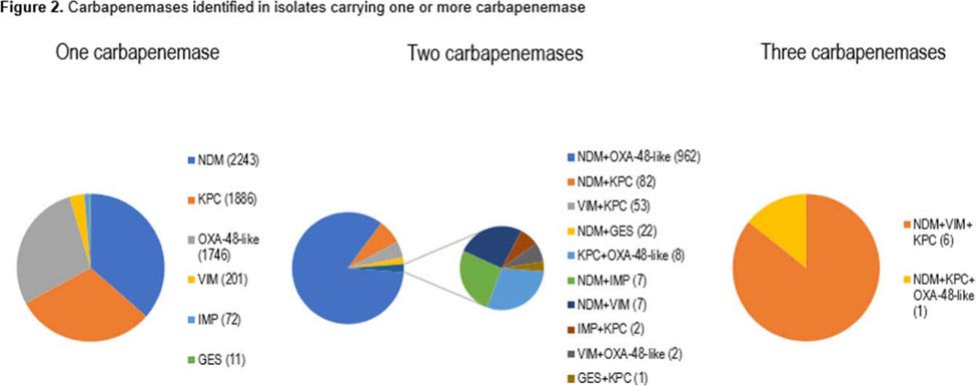

**Results:**

One or more carbapenemase gene was identified in 7312 isolates. In 2019, 10% of carbapenemase-positive isolates carried two carbapenemase genes, which increased to 21% by 2023 (Figure 1). Among isolates carrying two carbapenemase genes, the *bla_NDM_+bla_OXA-48-like_* genotype accounted for 84%, while *bla_NDM_+bla_KPC_* (7%), *bla_VIM_+bla_KPC_* (5%) and *bla_NDM_+bla_GES_* (2%) were the other most common genotypes (Figure 2). ATM-AVI was active *in vitro* against (genotype, percent susceptible): Single-carbapenemase positive, 97.7%; multiple-carbapenemase-positive, 98.1%; *bla_NDM_+bla_OXA-48-like_*, 98.0%; *bla_NDM_+bla_KPC_*, 98.8%; *bla_VIM_+bla_KPC_*, 98.1%, and *bla_NDM_+bla_GES_*, 100% (Table 1).

**Conclusion:**

Enterobacterales isolates that produce multiple carbapenemases are on the rise. While the NDM+OXA-48-like combination is still dominant, a plethora of combinations has been observed. While these organisms present complex resistance patterns, ATM-AVI demonstrated potent *in vitro* activity against them.

**Disclosures:**

Katherine Perez, PhD, Pfizer: Stocks/Bonds (Public Company)

